# Low value of routine serological screening for hepatitis B in patients with rheumatoid arthritis starting rituximab therapy in a nonendemic region

**DOI:** 10.1016/j.ero.2026.01.004

**Published:** 2026-02-02

**Authors:** Pauline M Bovens, Maike HM Wientjes, Nathan den Broeder, David F ten Cate, Aatke van der Maas, Alfons A den Broeder

**Affiliations:** 1 Department of Rheumatology, Sint Maartenskliniek, Ubbergen, The Netherlands; 2 Radboud Institute for Health Sciences, Radboud University Medical Centre, Nijmegen, The Netherlands; 3 Department of Research, Sint Maartenskliniek, Ubbergen, The Netherlands; 4 Department of Rheumatology, Radboud University Medical Centre, Nijmegen, The Netherlands

## Abstract

**Objectives:**

Rituximab (RTX) is an effective and safe treatment for rheumatoid arthritis (RA), but is associated with an increased risk of reactivation of hepatitis B virus (HBV) infection. Therefore, (inter)national guidelines recommend HBV screening prior to initiation of RTX treatment. However, the incidence of HBV in RA patients in nonendemic areas is expected to be very low, with known suboptimal screening adherence. This study aims to assess the added diagnostic value of routine serological HBV screening in RA patients initiating RTX.

**Methods:**

This retrospective single-centre cohort study included all patients with a clinical diagnosis of RA intending to initiate RTX between April 2012 and April 2024. Data on patient and disease characteristics, HBV screening results, and additional laboratory results were collected. Adherence to HBV screening, proportion of positive test results (hepatitis B surface antigen or core antibody positive), and incidence of HBV reactivation were assessed.

**Results:**

Of 975 included patients, 270 (28 %) were serologically screened for HBV. Six of those (2.2 %) had a positive screening result, of whom 3 eventually did not receive RTX. All 6 had a known HBV history or risk factor. No active HBV infections were observed after initiating RTX (mean follow-up 6.97 years), regardless of screening.

**Conclusions:**

Routine serological HBV screening identified very few previously unrecognised infections, and—in spite of screening adherence being low—no HBV reactivations occurred. Routine serological HBV screening, therefore, seems redundant in a low-risk population and should be reserved for patients with known risk factors.

**WHAT IS ALREADY KNOWN ON THIS TOPIC**

• Rituximab (RTX) increases the risk of hepatitis B reactivation in patients with chronic or resolved hepatitis B virus (HBV). Therefore, routine screening for HBV prior to the start of RTX is recommended by international guidelines. However, the added value of screening is unknown.

**WHAT THIS STUDY ADDS**

• In our low-risk population, no relevant unknown cases of HBV were found through routine serological screening, and no reactivation of HBV was observed. Therefore, the added diagnostic value of routine serological screening and the risk for HBV reactivation are very low in this population.

**HOW THIS STUDY MIGHT AFFECT RESEARCH, PRACTICE OR POLICY**

• Routine serological screening for HBV prior to the start of RTX treatment should not be done in low-risk populations and only in a few selected high-risk patients.

Alt-text: Unlabelled box dummy alt text

## INTRODUCTION

Rituximab (RTX), a monoclonal antibody directed against CD-20 B-cells, is widely used for the treatment of rheumatoid arthritis (RA). Although mostly considered a safe and effective treatment option, it is also associated with an increased risk of infections [1,2]. Among these infections is an elevated risk of hepatitis B virus (HBV) reactivation, especially in patients with chronic infections, although reactivation has also been observed in resolved HBV cases. It is therefore recommended by national and international guidelines to routinely screen for HBV prior to the start of RTX treatment and start antiviral prophylaxis if necessary [3].

However, the risk of HBV reactivation varies between patients and depends on multiple factors. First, in patients with a chronic HBV infection, the risk of reactivation is up to 80% [2,4,5], while in patients with a resolved infection, this risk is much lower, around 9% [6]. Second, most cases of HBV reactivation are known from patients with lymphoproliferative diseases, where RTX is part of combination therapy (Rituximab, Cyclosfosfamide, Hydroxydaunorubicine, Oncovin, Prednison (R-CHOP)). Therefore, a causal relationship between RTX and HBV reactivation is less certain, and the associated risk is expected to be lower in RA patients [4]. Furthermore, RA patients who start RTX have typically been treated with conventional disease-modifying antirheumatic drugs (DMARDs) for a long time, during which routine liver enzyme monitoring is standard practice. A previously undiagnosed infection would likely be detected then. As a result, the prevalence of undiagnosed active HBV infection in patients set to be treated with RTX is much lower. Overall, this makes the risk of HBV reactivation in these patients—particularly in countries with low HBV prevalence—almost negligible [7].

Although screening for HBV prior to the start of RTX and subsequent initiation of antiviral prophylaxis are recommended in current guidelines, adherence is generally suboptimal. Of note, adequate serological screening should include both hepatitis B surface antigen (HBsAg) and hepatitis B core antibody (anti-HBc). Prophylactic antiviral treatment is recommended for HBsAg-positive patients and patients with a resolved HBV infection (HBsAg-negative/anti-HBc positive) [3]. A Dutch study found that only 45.6% of patients were correctly screened for HBV before starting RTX treatment, while 30.2% had never been screened at all [8]. They also observed that among rheumatologists, the screening rates varied between 37% and 79%. Antiviral prophylaxis was started in 29.1% to 44.3% of patients at risk for reactivation. The question is whether this low protocol adherence is rational and justifiable in light of the limited risks.

The aim of this study is to evaluate the added diagnostic value of routine serological screening for HBV prior to the start of RTX in RA patients in a nonendemic country. This will be assessed by evaluating (1) adherence to the screening protocol, (2) the yield of screening for HBV, and (3) the incidence of HBV reactivation after start of RTX.

## METHODS

This retrospective cohort study was conducted in a large, multilocation, single rheumatology centre in the Netherlands, the Sint Maartenskliniek. All patients, aged 18 years or older, with a clinical diagnosis of RA who intended to start RTX treatment between April 2012 and April 2024 were included. HBV screening results of these patients and, in case of a positive screening result, risk factors for HBV were collected. Additional data on demographics, disease characteristics, laboratory results, and medication use, including previous and concurrent DMARDs, were retrospectively collected for all patients from the electronic health records (EHRs). Baseline was defined as the date of the first RTX infusion or HBV screening.

### Outcomes and measurements

For the first objective, the primary outcome was adherence to the protocol for HBV screening prior to the start of RTX treatment. The results from serological HBV screening included HBsAg, anti-HBc, and, when available, hepatitis B surface antibody (anti-HBs). Patients who had a screening result within 6 months before or after the start of RTX were classified as screened for HBV. All other patients were classified as not screened.

For the second objective, the primary outcome was the prevalence of unknown HBV infections in patients screened for HBV. Results from HBV screening were divided into 3 categories: positive (HBsAg+ and anti-HBc+), resolved infection (HBsAg− and anti-HBc+), or negative (HBsAg−, anti-HBc−, and anti-HBs−). In case of a positive or resolved infection, EHRs were evaluated for a known history of HBV infection and possible risk factors.

The third objective was to determine the risk of HBV reactivation, for which all patients receiving RTX were evaluated for signs of active HBV infection after the first RTX administration, with follow-up up to censoring (last known visit). Active HBV infection was defined as elevated ALT (Alanine Transaminase), more than 3 times above the upper limit, in combination with clinical signs or documentation of active HBV infection in the EHR.

### Statistical methods

Descriptive statistics were used to summarise the baseline characteristics and primary and secondary outcomes, reported as mean (+/−SD), median (IQR; Q1-Q3]), or frequency (percentage), depending on the distribution of the data. For point estimates of proportions, 95% CIs were calculated. No formal sample size calculation was made for this study, as the maximum convenient sample was included.

## RESULTS

### Participants

Between April 2012 and April 2024, 975 patients with RA were intended to receive RTX treatment. Of these, 3 patients did not receive RTX because of the screening result. Baseline characteristics of all patients are presented in Table 1.

**Table 1:** Patient and disease characteristics of all patients intended to receive RTX

	Overall N = 975 (100%)	Screened N = 270 (28%)	Not screened N = 705 (72%)
Female	694 (71%)	187 (69%)	507 (72%)
Age at start RTX (y)	61 (53, 69)	59 (50, 68)	62 (54, 70)
Disease duration (y)	8 (2, 16)	6 (2, 14)	9 (3, 16)
RF/ACPA positive	796 (84%)	210 (80%)	586 (86%)
ALT before RTX		24.5 (15.8)	25 (21)
No. of previous DMARDs		4.61 (2.44)	5.12 (2.31)
Concomitant DMARD use		269 (99.6%)	661 (93.8%)
Methotrexate		114 (42.2%)	246 (34.8%)
Leflunomide		50 (18.5%)	128 (18.2%)

RF: Rheumatoid Factor; ACPA: Anti-Citrullinated Protein Antibodies; ALT: Alanine Transaminase; DMARD, disease-modifying antirheumatic drug; RTX, rituximab.

n (%); median (IQR: Q1-Q3), mean (SD).

### Primary outcome: protocol adherence and results of HBV screening

Out of 975 patients intended to receive RTX, 270 (28%; 95% CI: 25%-31%) were screened for HBV prior to the start of RTX according to the protocol. Another 24 patients were screened for HBV more than 6 months before or after the first RTX infusion and, therefore, were considered as not being screened according to the protocol. There is significant variation between the treating physicians (*P* < .001) (Supplementary Fig).

Out of the 270 patients who were correctly screened, 6 had a positive test result (2.2%; 95% CI: 0.9%-5%). One patient had a known chronic HBV infection (HBsAg+ and anti-HBc+), which had already been diagnosed previously and therefore did not receive RTX. Five patients had a serologic previous infection with HBV (HBsAg- and anti-HBc+). Three were born in endemic countries, of whom 1 initiated RTX without antiviral prophylaxis, while the other 2 patients from endemic countries did not initiate RTX. The 2 other patients were known carriers of HBV, and both received RTX without antiviral prophylaxis (Table 2).

**Table 2:** Details of patients with positive serological screening

Patient	HBsAg	Anti-HBc	Infection	Risk factor	RTX	Antiviral prophylaxis
1	Positive	Positive	Chronic	Known carrier of HBV	No	-
2	Negative	Positive	Resolved	Born in an endemic country	No	-
3	Negative	Positive	Resolved	Born in an endemic country	No	-
4	Negative	Positive	Resolved	Born in an endemic country	Yes	No
5	Negative	Positive	Resolved	Known carrier of HBV	Yes	No
6	Negative	Positive	Resolved	Known carrier of HBV	Yes	No

Anti-HBc, hepatitis B core antibody; HBsAg, hepatitis B surface antigen; HBV, hepatitis B virus; RTX, rituximab.

### HBV reactivation

To identify HBV reactivation, ALT levels were screened for elevation more than 3 times the upper limit of normal. At least 1 ALT measurement after RTX initiation was available for 938 patients with a mean follow-up duration of 6.97 patient years (SD 3.87) and a median of 15 measurements per patient (IQR 7-26). ALT measurements were primarily missing because the patient did not have a follow-up visit due to the recent start of RTX. Elevated ALT was found in 64 patients (7.1%; 95% CI: 5.6%-9.0%) from whom 30 were in the first year after the start of RTX. There were no cases observed of active HBV infections or reactivation, either in patients screened or not screened.

## DISCUSSION

This study examined the protocol adherence to screening for HBV and the outcome of screening prior to the start of RTX in a low-incidence region. Screening rates were low, and when done, they rarely revealed unexpected cases of HBV. Furthermore, no reactivation of HBV was observed in this population up to a mean of 7 years after starting RTX treatment, regardless of screening or antiviral prophylaxis.

The screening adherence rate of 28% is slightly lower but comparable to what is found in the literature; for example, Brakenhoff et al [8] reported a screening rate of 37% among rheumatologists in nonacademic hospitals in the Netherlands. Protocol adherence in medicine in general varies widely and is often within the range we report. However, this suboptimal screening adherence provided an opportunity to assess the risk of HBV reactivation in unscreened individuals starting RTX.

The results from RTX-associated HBV screening are also consistent with findings from other studies in nonendemic countries. A study in a South Australian population found 1.4% HBsAg-positive cases in the screened population and 3.4% resolved infections [9]. Another Dutch study found 0.5% HBsAg-positive cases and 5.1% resolved infections [8]. Of note, both studies also included patients with other indications for RTX than RA. Furthermore, we observed no signs of HBV reactivation, which is in line with other studies in RA patients [7,9]. It is notable that patients with a positive screening result did not receive antiviral prophylaxis before starting RTX, even after consulting an infectiologist or gastroenterologist, which questions the very rationale for screening. However, this also did not result in reactivation of HBV.

There are a few limitations to this study. Firstly, there might be a selection bias in deciding who to screen. We found that this is mainly physician dependent, as some rheumatologists are more protocol-adherent than others, and patient characteristics are very comparable between screened and not-screened patients. Furthermore, high-risk patients or patients with known HBV might not even be considered for RTX treatment and are thus not part of the study population. Secondly, only elevated liver enzymes and clinical data were evaluated to find possible reactivation, while no data were available about HBV DNA levels. This might lead to underestimating the number of reactivations. However, we assume that clinically relevant HBV reactivation is very unlikely in light of an average of 7 years of ALT and clinical monitoring in a high-income health care system. Thirdly, this study was conducted in a single centre—albeit with 10 hospital locations—in the central, south, and eastern part of the Netherlands, regions with a low prevalence of HBV. Therefore, the results should be interpreted within this context.

The potential added value of screening lies in its ability to provide information on current HBV status, which may lead to changes in clinical management. This requires a relevant a priori chance of detecting previously unknown HBV and acting upon this information by changing therapy, starting antiviral prophylaxis, or closely monitoring the patient for possible HBV reactivation. The current EULAR (European Alliance of Associations for Rheumatology) guideline recommends screening all patients starting with a biological DMARD in order to identify patients who might benefit from antiviral prophylaxis, mainly HBsAg-positive patients [3,10]. Additionally, it is suggested that all patients starting RTX should be referred to a gastroenterologist because they are at high risk for HBV reactivation [3]. However, our study shows that this advice might be excessive in a population where the prevalence of HBV is very low, let alone unknown HBV. Screening should therefore be reserved for patients with a high risk of HBV who have not been recently tested. Known risk factors and at-risk populations for (chronic) HBV include a history of injection drug use, multiple sexual partners, individuals from high-prevalence countries (East and Southeast Asia, Sub-Saharan Africa) who have not been screened previously, and health care professionals (when not vaccinated). The main risk factor in our study is being born in an endemic country. In order to achieve successful, risk-based screening, it is important to educate rheumatologists about these risk factors, since previous studies stated that it can be difficult to recognise patients at risk [11].

### Conclusion

In conclusion, routine serological screening for HBV in all RA patients starting RTX treatment seems redundant in a low-risk population, and HBV screening should be reserved for patients with known risk factors.

## CRediT authorship contribution statement

**Pauline M Bovens:** Writing – review & editing, Writing – original draft, Methodology, Formal analysis, Conceptualization. **Maike HM Wientjes:** Writing – review & editing, Methodology, Conceptualization. **Nathan den Broeder:** Writing – review & editing. **David F ten Cate:** Writing – review & editing. **Aatke van der Maas:** Writing – review & editing. **Alfons A den Broeder:** Writing – review & editing, Methodology, Conceptualization.

## Competing interests

All authors declare they have no competing interests.

## Appendix Supplementary materials

Supplementary figure: Proportion of patients screened per physician Image, application 1
