# Supplementary figures and images for: Low value of routine serological screening for hepatitis B in patients with rheumatoid arthritis starting rituximab therapy in a nonendemic region
Source: EULAR Rheumatol Open. 2026 Feb 2;2(1):199–202. doi: 10.1016/j.ero.2026.01.004 (PMC13292407; doi:10.1016/j.ero.2026.01.004)

Supplemental data

Supplementary figure: Proportion of patients screened per physician


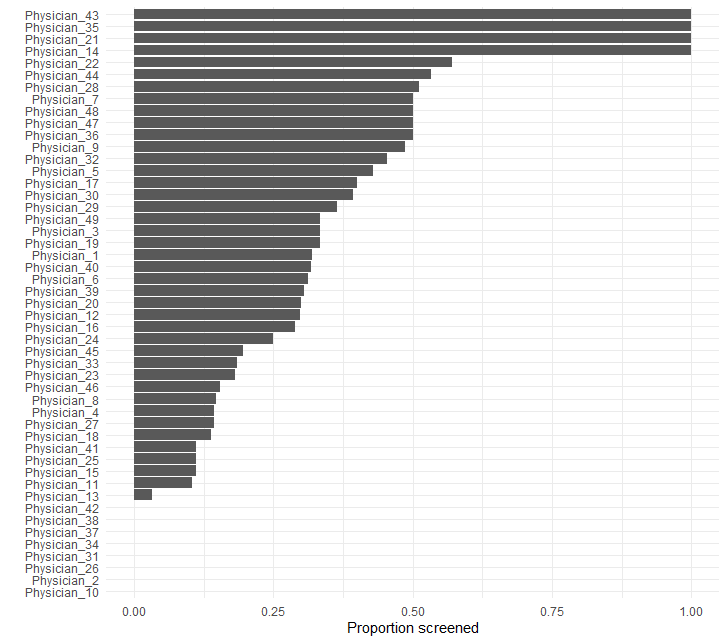

Supplement: Supplementary file 1 — Supplementary figure: Proportion of patients screened per physician [file mmc1.docx]
